# Aerobic Interval Training Prevents Age-Dependent Vulnerability to Atrial Fibrillation in Rodents

**DOI:** 10.3389/fphys.2018.00206

**Published:** 2018-03-09

**Authors:** Vegard Malmo, Allen Kelly, Karin S. Garten, Tomas Stolen, Natale P. L. Rolim, Ulrik Wisloff, Godfrey Smith, Jan P. Loennechen

**Affiliations:** ^1^K.G. Jebsen Center of Exercise in Medicine, Department of Circulation and Medical Imaging, Norwegian University of Science and Technology, Trondheim, Norway; ^2^Clinic of Cardiology, St. Olavs Hospital, Trondheim, Norway; ^3^Institute of Cardiovascular and Medical Sciences, University of Glasgow, Glasgow, United Kingdom; ^4^School of Human Movement and Nutrition Sciences, The University of Queensland, Brisbane, QLD, Australia

**Keywords:** atrial fibrillation, exercise, aging, conduction velocity, fibrosis

## Abstract

**Aims:** Increasing age is the most important risk factor for atrial fibrillation (AF). Very high doses of exercise training might increase AF risk, while moderate levels seem to be protective. This study aimed to examine the effects of age on vulnerability to AF and whether long-term aerobic interval training (AIT) could modify these effects.

**Methods:** Nine months old, male Sprague-Dawley rats were randomized to AIT for 16 weeks (old-ex) or to a sedentary control group (old-sed), and compared to young sedentary males (young-sed). After the intervention, animals underwent echocardiography, testing of exercise capacity (VO_2max_), and electrophysiology with AF induction before *ex vivo* electrophysiology. Fibrosis quantification, immunohistochemistry and western blotting of atrial tissue were performed.

**Results:** Sustained AF was induced *in vivo* in 4 of 11 old-sed animals, but none of the old-ex or young-sed rats (*p* = 0.006). VO_2max_ was lower in old-sed, while old-ex had comparable results to young-sed. Fibrosis was increased in old-sed (*p* = 0.006), with similar results in old-ex. There was a significantly slower atrial conduction in old-sed (*p* = 0.038), with an increase in old-ex (*p* = 0.027). Action potential duration was unaltered in old-sed, but prolonged in old-ex (*p* = 0.036). There were no differences in amount of atrial connexin 43 between groups, but a lateralization in atrial cardiomyocytes of old-sed, with similar findings in old-ex.

**Conclusion:** AF vulnerability was higher in old-sed animals, associated with increased atrial fibrosis, lateralization of connexin-43, and reduced atrial conduction velocity. AIT reduced the age-associated susceptibility to AF, possibly through increased conduction velocity and prolongation of action potentials.

## Introduction

Atrial fibrillation (AF) is the most common sustained arrhythmia in humans, with an estimated prevalence of 2–3% in the adult population. It is associated with increased morbidity and mortality, mainly due to embolic stroke and associated comorbidities (Kirchhof et al., 2016). AF symptoms reduce quality of life, and AF treatment imposes a major cost on health-care services (Andrade et al., 2014).

Focal ectopic firing, caused by abnormal Ca^2+^ handling and changes in autonomic nerve activity and response, is the common trigger of AF episodes. However, this will usually not cause sustained AF in the healthy atria. Structural and/or electrical changes in the left atrium (LA) are necessary for sustained AF to occur. The most common electrophysiological mechanisms maintaining AF are re-entry and rotor activity, and less commonly rapid ectopic firing (Andrade et al., 2014; Heijman et al., 2014).

Age is the most important risk factor for AF, causing an accelerating increase in prevalence from about 60 years of age (Andrade et al., 2014). Due to an aging population, the prevalence of AF is rising worldwide. The mechanisms underlying the higher susceptibility to AF with aging are not clear. Probable mechanisms are structural changes with atrial dilatation and fibrosis, and abnormalities in Ca^2+^ handling with advancing age. Electrical remodeling with changes in properties of connexins, such as changes in phosphorylation state, reduced expression and/or changes in distribution of connexins on the cell surface of cardiomyocytes also occur with aging (Sankaranarayanan et al., 2013). These factors can increase the susceptibility to AF through general and local slowing of conduction velocity (CV) and shortening and spatial dispersion of action potential duration (APD).

The effect of exercise on age-induced AF susceptibility is uncertain. There is a higher risk of AF among athletes after high volumes of endurance exercise over years (Mont et al., 2009). This has also been shown in animal studies, pointing at autonomic changes with increased parasympathetic influence on the atrium, and structural changes with atrial dilatation and fibrosis as probable mechanisms (Mont et al., 2009; Guasch et al., 2013; Aschar-Sobbi et al., 2015). The amount of exercise that is detrimental is not known, but exercise induced AF is mainly associated with high endurance sports activity performed from younger age. There is emerging evidence of a protective effect on AF risk by low and moderate levels of physical activity (Drca et al., 2014), and cardiorespiratory fitness has been shown to have an inverse relationship to AF risk (Qureshi et al., 2015). Modification of risk factors for AF reduced AF burden and improved outcome after radiofrequency ablation (Pathak et al., 2014). Aerobic interval training (AIT) also reduced the burden of AF in patients with non-permanent AF (Malmo et al., 2016). This was a relatively healthy AF population with few risk factors and the effect was apparent within a few weeks of the exercise intervention, which points to more direct effects of exercise on AF than through modification of AF risk factors.

Exercise and especially AIT has favorable effects on several of the processes underlying AF. It can improve Ca^2+^ handling, reduce inflammation, reverse unfavorable electrical remodeling, and induce positive autonomic cardiovascular changes (Johnsen et al., 2013; Rodrigues et al., 2014). The effects on atrial electrophysiological properties and the mechanisms underlying a positive effect of exercise in AF are largely unknown.

The aim of the current study was to examine the effects of aging on AF susceptibility in middle-aged rats, whether AIT would modify these effects and to suggest possible underlying mechanisms for age- and exercise induced changes.

## Materials and methods

Nine months old male, Sprague-Dawley rats arrived in the same batch and were randomly assigned to AIT (older exercise; old-ex) for 16 weeks or a sedentary control group (older sedentary; old-sed); *n* = 16 in each group. Rats were kept in the same housing room during the study period. Four months old rats (*n* = 12) were included as a young sedentary control group (younger sedentary; young-sed) (All rats from Harlan Laboratories, now Envigo, The Netherlands). After the 16-week intervention period, *in vivo* electrophysiology and echocardiography were performed at separate time points, 24–48 h after the last bout of exercise. To be certain that the *in vivo* electrophysiology procedure did not affect *ex vivo* measurements, we waited at least 7 days before rats were consecutively euthanized and hearts immediately brought to *ex vivo* experiments with Langendorff perfusion. Animals continued exercise in this 7-day period. Hearts were then cut down; the four cardiac chambers quickly separated, dissected free from surrounding structures and cut in two longitudinally, with one half snap-frozen in liquid nitrogen and the other preserved by 4% paraformaldehyde fixation. *Ex vivo* experiments were performed on 21 randomly chosen animals (6 young-sed, 7 old-sed, 8 old-ex) Investigators performing experiments and analyses were blinded to the intervention group. This study was carried out in accordance with the recommendations of National Regulations, the European Convention for the Protection of Vertebrate Animals used for Experimental and other Scientific Purposes (ETS No.123), and the European Directive 2010/63/EU on the protection of animals used for scientific purposes. The protocol was approved by the Norwegian Animal Research Authority. The general health of the animals was monitored by an independent veterinarian.

### Testing of maximal oxygen uptake and exercise training

Maximal oxygen uptake (VO_2max_) testing was performed with the animals running until exhaustion on a treadmill at 25° inclination in a metabolic chamber, as previously described (Wisloff et al., 2001). Exercise rats performed 1 h of AIT on treadmills with separate lanes and 25° inclination, five consecutive days a week for 16 weeks. The protocol for each session was 10 min of warming up at ~60% of VO_2max_, followed by eight 4-min intervals of uphill treadmill walking or running at high intensity (estimated 80–90% of VO_2max_) with 2 min of active rest between intervals and a 4-min active cool-down period at the end. The inclination of the treadmill was kept constant during the study, while speed was increased gradually as the exercise capacity of the rats increased. All animals were scored each session, according to a running score from 0 to 5 to monitor exercise effort (Guasch et al., 2013).

### Anesthesia

All invasive procedures were performed under general anesthesia. Anesthesia was induced by inhalation of 5% isoflurane and O_2_ in a closed chamber, and was maintained by inhalation of 1.5% isoflurane and O_2_. Rats received a weight-adjusted injection of buprenorphine before surgical procedures.

### *In vivo* electrophysiology

Animals were placed on a heated electrocardiogram (ECG)-board, for continuous recording of ECG. A rectal thermometer was inserted to ensure a stable temperature at 37°C. A 1.6 Fr octapolar catheter with 1 mm inter-electrode distance (Millar Instruments Inc., Houston, Texas, USA) was inserted in the right cervical vein and placed in the right atrium (RA). Intracardiac and surface ECG-signals were recorded by Iox software, and later analyzed by the same program, in addition to Ecg-auto (both EMKA Technologies, Paris, France). Pacing was performed at twice the current threshold. Inducibility of AF was tested by atrial burst trains at a cycle length (CL) of 60, 40, and 20 ms for 7.5, 15, and 30 s respectively, with one burst train at each point. AF was defined as more than 1 s of irregular atrial electrograms at >800 bpm with irregular ventricular response. Episodes of AF lasting more than 30 s were considered sustained (Guasch et al., 2013).

### Echocardiography

Echocardiography was performed using a Vevo 2100 system (VisualSonics, Ontario, Canada) with a 24-MHz transducer in the left parasternal position. Examinations were performed in the supine position on a heating platform to maintain a body temperature at 37°C. Left atrium dimension (LAD) was measured in long axis view at the end-systole, with an M-mode cursor line placed through the left atrium walls at the level of the aortic valves, with two-dimensional guidance. Left ventricle (LV) wall thickness and cavity diameters were measured in short axis view, both in end-diastole and end-systole, with the M-mode cursor perpendicular to the LV anterior and posterior walls, at the level of the papillary muscles. Ejection fraction (EF) was calculated from cavity diameters. To estimate LV mass and LV volume, the area-length method was used. Doppler recordings were obtained from the apical 4 chamber view by positioning the Doppler sample volume parallel to flow direction, and mitral valve (MV) early wave (E) peak and MV atrial wave (A) peak were measured and MV E/A ratio calculated. Myocardial velocities were recorded at the level of basal septal segment from the apical view using pulsed Doppler tissue imaging. Early (E') and late (A') diastolic waves were measured, and E/E' ratio was calculated. All measurements were performed excluding the respiration peaks and obtained in triplicate. As cardiac dimensions are closely related to body size, all parameters, except Doppler measurements and ratios were normalized to weight (Guasch et al., 2013).

### Conduction velocity measurements and optical mapping

After excision, hearts were immediately placed in ice cold Tyrode's solution, comprising (mM): NaCl (120.9), KCl (5.9), MgSO_4_ (1.0), Na_2_HPO_4_ (1.0), NaHCO_3_ (24.8), CaCl_2_ (1.8) and glucose (15.0), oxygenated with a 95% O_2_/5% CO_2_ gas mixture (constant pH 7.4). Hearts were then cannulated via the ascending aorta and retrogradely perfused with Tyrode's at 37°C. Blebbistatin (7.5 μM) was added to remove mechanical activity. Pacing was performed at twice the diastolic threshold with 2 ms pulses, via bipolar electrodes on the right and left atrial appendages respectively, with an average pacing frequency of 5.5 Hz. Conduction velocity was measured in paced hearts with a custom-made cylindrical electrophysiology probe (CV probe). The probe contained 3 pairs of Ag/AgCl electrodes; 1 pair delivered a stimulus pulse to activate the cardiac tissue, while the 2 remaining distal pairs, set at a known distance from each other recorded the subsequent activation waveform as it passes across each pair, visualized as a rapid electrical deflection. Conduction velocity was calculated by dividing the distance between the recording electrode pairs (2 mm) by the time delay between the electrical deflections recorded by each electrode pair. For optical action potential (AP) recordings, hearts were loaded with the potentiometric fluorophore di-4 ANEPPS (10 μM stock concentration) over a 10-min period by slow injection of 50 μl dye into a bubble trap proximal to the aortic cannula and APs measured from the epicardial surface of the RA and LA using a light guide system (Cairn Research, Faversham, UK). Hearts were electrically challenged using a ramped pacing protocol (6–22 Hz for 1 min in 2 Hz intervals). After the end of experiments, a 5-min standardized pacing protocol (RA pacing at 6 Hz) was applied to all hearts, to limit individual differences caused by pacing in later western-blot and immunohistochemistry (IHC) analyses.

### Antibodies

Polyclonal antibodies against connexin 43 (cx-43) (C6219, Sigma-Aldrich), connexin 40 (sc-20466, Santa Cruz Biotechnology Inc.; AB-1726, Chemicon/Merckmillipore; and ACC-205, Alomone labs), Nav1.5 (ASC-005, Alomone labs), and Ser 368 (SC-101660) and Ser 279/282 (sc-12900) phosphorylated cx-43 (both Santa Cruz Biotechnology, Inc.) were used as primary antibodies. Antibodies were tested with different solutions, from recommendations in data sheet from the suppliers, but only the anti cx-43 (C6219 SIGMA) and anti cx-43 (Ser 368) (SC-101660) gave acceptable results, and were used in western blot analyzes and immunohistochemistry. Cx-40 has previously been shown to be scarcely expressed in rat atria compared to other species (Iwasaki et al., 2014). IRDye 800CW goat anti-rabbit IgG (Licor) was used as secondary antibody.

### Western-blot

The frozen tissue was homogenized in lysis buffer, 50 mM Tris pH-7.5, 150 mM NaCl, 0.5% NP-40, 1 mM EDTA, complete protease inhibitor (Roche) and phosphatase inhibitor cocktail 2 and 3 (SIGMA), in a MAGNALyser and 1.4 mm zirconium beads using 4^*^30 s 5000 rpm cycles. The soluble fraction was collected after centrifugation and protein concentration measured. Gel electrophoresis (Novex, Invitrogen) and blotting to a nitrocellulose membrane were done using 50 μg of protein extract. Western analyses were performed using primary antibodies described above, secondary antibodies were from Licor, and the membranes were scanned on an Odyssey Licor IR imager. Anti-Connexin-43 was used at 1:3000 dilution, and anti-connexin-43 Ser 368 at 1:200.

### Immunohistochemistry and fibrosis quantification

Atrial tissue was fixed in fresh phosphate buffered 4% paraformaldehyde (PFA) at 4°C overnight and subsequently stored in 0.4% PFA at 4°C prior to paraffin embedding. For fibrosis quantification, deparaffinized tissue sections were stained with Masson's Trichrome. For immunohistochemistry (IHC), 4 μm thick FFPE tissue sections were mounted on Superfrost+ glass slides and dried at 37°C overnight. The slides were preheated at 60°C for 90 min and deparaffinized before heat-induced epitope-retrieval (HIER) were performed using DAKO Target Retrieval Solution (K8004) at 97°C for 20 min in a DAKO PT Link pre-treatment module (Dako, Glostrup, Denmark). Immunohistological staining was performed using a DAKO Autostainer and DAKO EnVision+ HRP Detection System with diaminobenzidine (DAB+) chromogene (K4011). The sections were incubated with DAKO Protein block (X0909) for 20 min followed by immunostaining with anti-Connexin 43 (1:500) for 40 min. Sections were counterstained with hematoxylin. The IHC stained sections were examined by an authorized pathologist. Images were acquired using an Olympus BX41 microscope with an Olympus DP25 camera and CellB Imaging software (Olympus Soft Imaging Solutions) and further analyzed using ImageJ 1.49 g (NIH, Bethesda, MD). For fibrosis quantification, the whole tissue section was photographed with at least 25 images per sample. A macro validated for Picrosirius-red stained tissue was used, (Hadi et al., 2011) with adjustments made to fit the Massons's staining. Endocardial, epicardial, and perivascular fibrosis were manually excluded from analyses. For IHC, areas with longitudinal cardiomyocyte orientation were imaged; at least 5 per sample. The amount of connexin-43 at the intercalated disc and lateral side respectively were then quantified manually.

### Statistical analysis

Continuous, normally distributed variables are presented as mean ± standard error of the mean (SEM) to improve visual clarity of figures. Non-normally distributed data are presented as median ± interquartile range. Differences in the primary endpoint of AF inducibility were compared using the Fisher exact test. To examine the effects of age and exercise respectively, univariate ANOVA analyzes were performed for normally distributed data, with age and exercise as main effects. Kruskal-Wallis and Mann-Whitney *U*-tests were used for non-normally distributed data. Analyses were performed using SPSS Statistics 21 (IBM SPSS Statistics, IBM Coroporation, Amonk, New York) and GraphPad Prism 6 (GraphPad Software, Inc., La Jolla, California). All tests were performed with two-sided *p*-values, and *p*-values below 0.05 were considered statistically significant.

## Results

### Study protocol adherence

Two animals in the old-ex group and five in the old-sed group were euthanized between study week 7 and *in vivo* electrophysiology due to intercurrent illnesses. No specific disease was detected by post-mortem examination. Thus, 12 young-sed, 11 old-sed, and 14 old-ex animals underwent *in vivo* electrophysiology. One young-sed animal died at the end of electrophysiology due to cardiac perforation during removal of the catheter, and 2 old-sed and 5 old-ex had to be euthanized before echocardiography. Of those, 21 animals were further randomly selected for *ex vivo* experiments, with 6 from young-sed, 7 old-sed, and 9 form the old-ex group. Of those, 3 had sustained AF, 7 non-sustained AF, and 10 had no AF during *in vivo* electrophysiology. The probes were too large for placement on some atria, which was especially a problem for the LA. All measurements are therefore not available for all animals. The number of animals included in each analysis is specified in the figure legends.

All animals were put on the treadmills 5 days a week for the whole study period, but not all animals were able to follow the exercise protocol with adequate intensity and duration fully throughout the whole intervention period, with 4, 7, and 9 rats completing less than respectively 70, 80, and 90% of the exercise sessions with a running score of 3 or more. No animals were however excluded from the study due to low exercise effort, and all animals were included in analyses.

### Exercise capacity and cardiac chamber morphology

**Figure 1:**
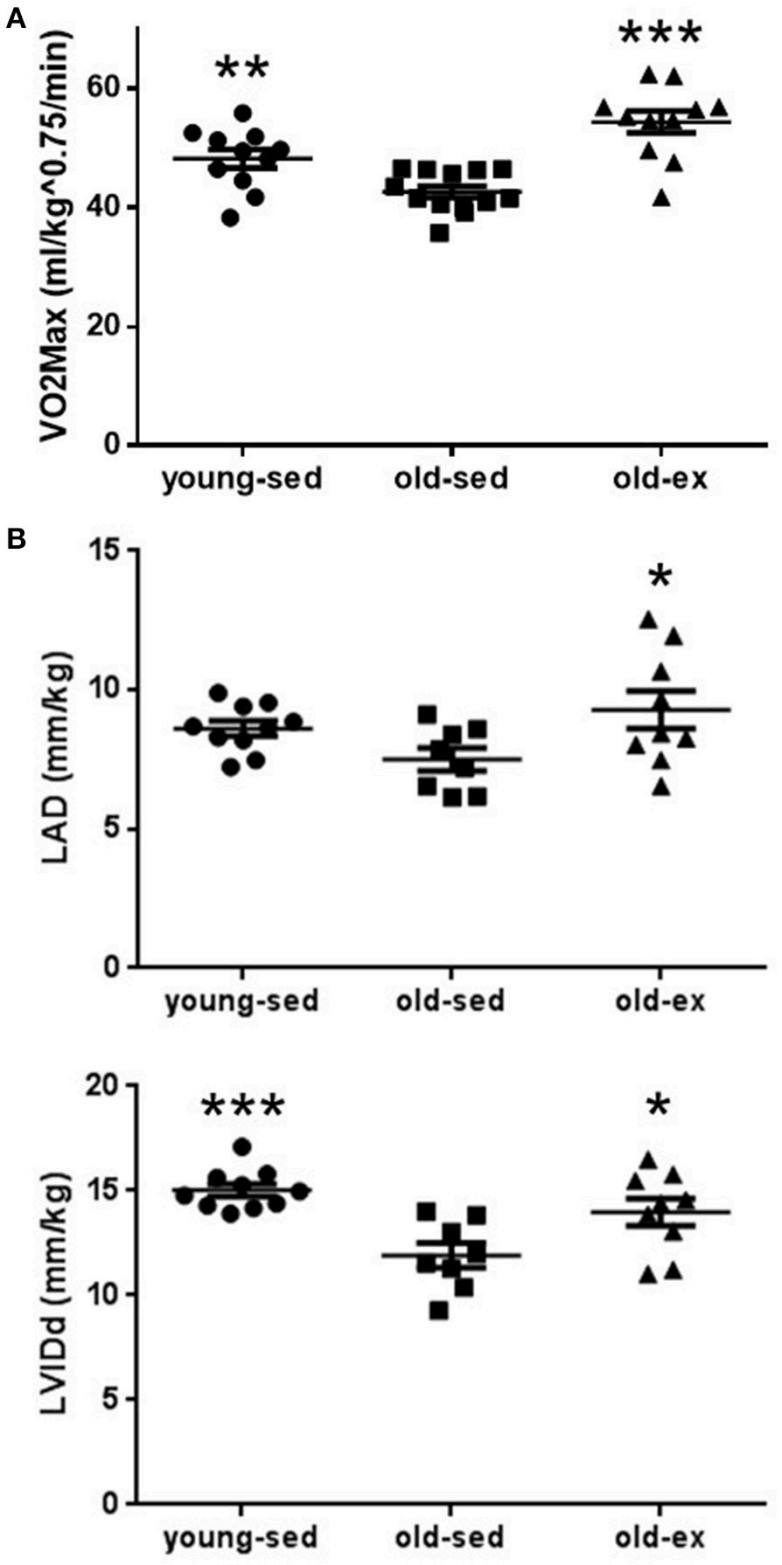
Exercise capacity and cardiac chamber size measured after the 16 week intervention period**. (A)** Maximal oxygen uptake. **(B)** Echocardioraphic measurements of: Upper: Left atrial diameter. Lower: Left ventricular internal diameter in diastole. LAD, Left atrial diameter; LVIDd, Left ventricular internal diameter in diastole; Old-ex, Older exercised animals; Old-sed, Older sedentary animals; Young-sed, Younger sedentary animals; VO_2_max, Maximal oxygen uptake. *p < 0.05; **p < 0.01; ***p < 0.001; vs. old-sed.

AIT eliminated the age-related drop in exercise capacity (Figure 1A). VO_2max_ was significantly lower in old-sed (*p* = 0.007), with a significant increase in old-ex (*p* < 0.0001). Sixteen weeks of training also altered cardiac morphological parameters as assessed by echocardiography, summarized in Table 1. As there was a significant effect of age and exercise on weight, all parameters, except Doppler measurements and ratios were normalized to weight. There was a significant decrease in left ventricular diastolic diameter in old-sed compared to young-sed when normalizing to weight, and a trend toward a reduced ejection fraction (EF). The left atrial diameter (LAD) was increased in absolute terms in old-sed, but this was not significant when normalized to body weight. Exercise increased LAD and ventricular diameters compared to old-sed when normalized to body weight (Figure 1B).

**Table 1:** Echocardiographic results.

	**Young-sed**	**Old-sed**	**Old-ex**
Weight, g	550 ± 8.9	700 ± 22.4^###^	627 ± 18.3^*^
E, cm/s	924 ± 90	779 ± 77	726 ± 53
E/A ratio	1.4 ± 0.09	1.5 ± 0.17	1.4 ± 0.11
LVAWd, mm	1.93 ± 0.06	2.36 ± 0.08^###^	2.19 ± 0.09
LVAWd, mm/kg	3.48 ± 0.09	3.40 ± 0.04	3.52 ± 0.20
LVIDd, mm	8.33 ± 0.13	8.41 ± 0.25	8.72 ± 0.33
LVIDd, mm/kg	15.03 ± 0.30	11.91 ± 0.6^###^	13.98 ± 0.64^*^
LVIDs, mm	4.70 ± 0.16	5.34 ± 0.25^#^	5.77 ± 0.33
LVIDs, mm/kg	8.49 ± 0.34	7.49 ± 0.47	9.26 ± 0.61^*^
LVPWd, mm	2.27 ± 0.11	2.17 ± 0.09	2.29 ± 0.11
LVPWd, mm/kg	4.09 ± 0.19	3.20 ± 0.16^##^	3.67 ± 0.20
EF, %	72 ± 2.2	64 ± 3.4^§^	61 ± 2.7
LV Mass, g	1.47 ± 0.06	1.69 ± 0.04^##^	1.74 ± 0.09
LV Mass g/kg	2.79 ± 0.16	2.43 ± 0.11	2.79 ± 0.16
LV mass, corr, g	1.2 ± 0.04	1.36 ± 0.03^##^	1.40 ± 0.07
LV mass, corr, g/kg	2.12 ± 0.07	1.95 ± 0.08	2.24 ± 0.13
e', m/s	60 ± 4	63 ± 5	56 ± 5
E/e'	16 ± 2	13 ± 2	13 ± 1
LAD, mm	4.78 ± 0.15	5.39 ± 0.26^§^	5.77 ± 0.36
LAD, mm/kg	8.62 ± 0.27	7.50 ± 0.41	9.29 ± 0.69^*^

Values are mean ± SEM. A, Transmitral peak velocity during atrial systole; E, Transmitral peak velocity of early filling; e', Peak annular velocity in early myocardial relaxation; LAD, Left atrial diameter; LV, Left ventricle; LVIDd, Left ventricular diameter in diastole; LVAWd, Left ventricular anterior wall diameter in diastole; LVPWd, Left ventricular posterior wall diameter in diastole; LVIDs, Left ventricular diameter in systole; EF, Ejection fraction; Old-sed, Older sedate; Old-ex, Older exercise; Young-sed, Younger sedate. For age:

§ p = 0.05

# p < 0.05

## p < 0.01

### p < 0.001. For exercise:

* p < 0.05

^**^p < 0.01 ^***^p < 0.001.

### AF vulnerability

**Figure 2:**
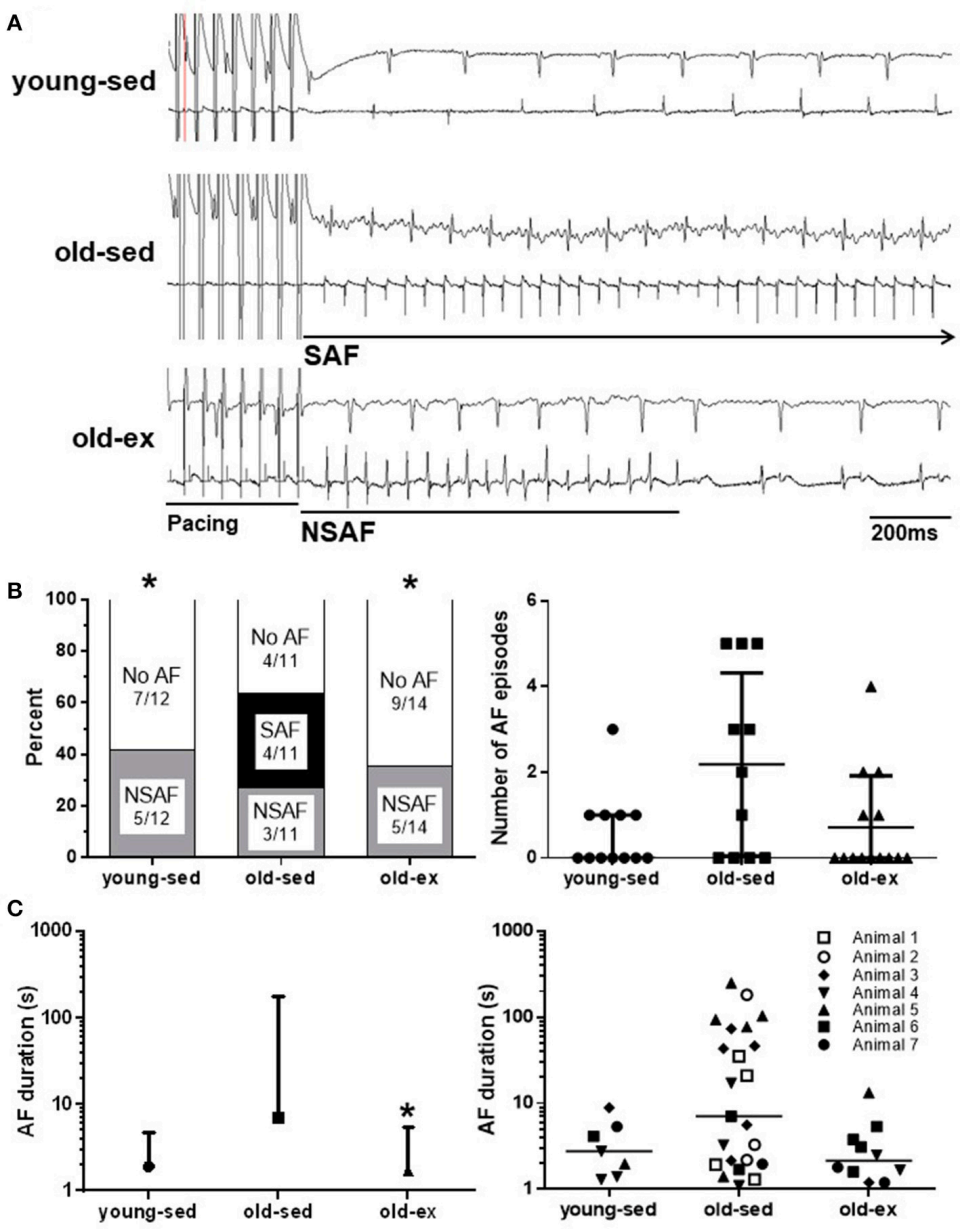
AF vulnerability after the 16-week intervention period. AF vulnerability measured by *in vivo* electrophysiology after the 16-week intervention period. **(A)** Surface ECG (top) and intra-atrial ECG (bottom) recordings from *in vivo* electrophysiology in; Upper: Young-sed with SR. Middle: Old-sed with sustained AF. Lower: Old-ex with non-sustained AF. **(B)** Left: Inducibility of AF by *in vivo* electrophysiology. Right: Number of AF induced episodes per animal. **(C)** Left: Duration of the longest AF episode in each animal. Right: Duration of all recorded AF episodes for all animals, showing intra-group variation. Number refers to different animals in each group. AF, Atrial fibrillation; ECG, electrocardiogram; NSAF, Non-sustained AF; SAF, Sustained AF; SR, Sinus rhythm. *p < 0.05 vs. old-sed. Error bars are only shown one-sided where crossing 0.

Whether exercise training interacts beneficially with the increased AF susceptibility seen with aging is unclear. To test this, animals were subjected to electrophysiological pacing protocols *in vivo*. Typical ECG recordings are shown in Figure 2A. Sustained AF was induced in 4 of the 11 old-sed animals, but in none of the young-sed (*p* = 0.017 vs. old-sed) or old-ex animals (*p* = 0.012 vs. old-sed) (Figure 2B—left). In old-sed, there was a trend toward a larger number (*p* = 0.082) and longer duration of AF episodes compared to young-sed (*p* = 0.13 for all episodes and *p* = 0.087 for the longest episode) (Figure 2B—right and Figure 2C). The duration of AF episodes was shorter after AIT (*p* = 0.031 for all episodes and *p* = 0.040 for the longest episode compared to old-sed) (Figure 2C).

### Atrial conduction and action potential measurements

**Figure 3:**
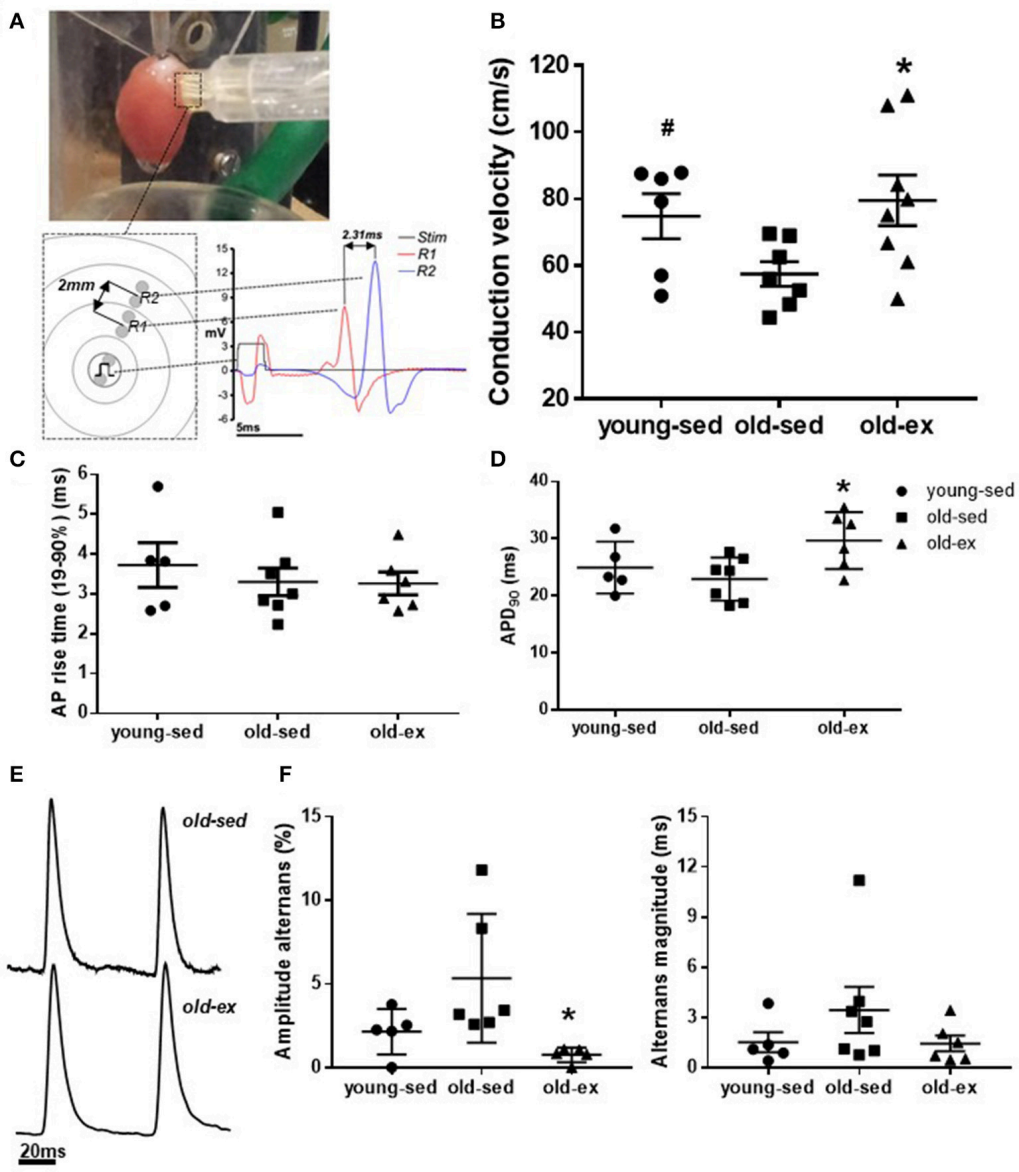
*Ex vivo* electrophysiology after the 16-week intervention period. **(A)** CV probe on isolated perfused heart, and example recording from CV measurements. **(B)** Right atrial CV (young-sed *n* = 5, old-sed *n* = 7, old-ex *n* = 6). **(C)** AP rise time (10-90%) measured in RA at a cycle length of 175 ms (young-sed *n* = 5, old-sed *n* = 7, old-ex *n* = 6). **(D)** APD at 90% repolarization at a cycle length of 175 ms for right atrium (young-sed *n* = 5, old-sed *n* = 7, old-ex *n* = 6). **(E)** Example AP recordings of old-ex and old-sed animals. **(F)** AP amplitude alternans (left) and APD alternans magnitude (right) at a stimulation cycle length of 60 ms in the right atrium. APD, Action potential duration; CL, Cycle length; CV, Conduction velocity. *p < 0.05 vs. old-sed.

The reduction in AF susceptibility in the old-ex group may result from structural and/or functional remodeling of atrial tissue. To investigate this, a series of electrophysiological measurements were performed to establish functional changes at the tissue level. Figure 3A shows placement of a CV probe during a typical experiment, with the mechanism of operation and example traces displayed diagrammatically below. CV data for each group are shown in Figure 3B. CV was significantly lower in old-sed compared to young-sed (*p* = 0.038), while it was increased in old-ex compared to old-sed (*p* = 0.027). Optical AP recordings revealed that there was no change in APD with age, but a significant increase in exercised animals at baseline CLs (175 ms) (Figures 3C,D). At shorter CLs (60 ms), significantly lower AP amplitude- and duration alternans were apparent in the old-ex animals, compared to old-sed (Figures 3E,F). AP rise time (10–90%) was the same in all groups (Figure 3).

### Atrial fibrosis

**Figure 4:**
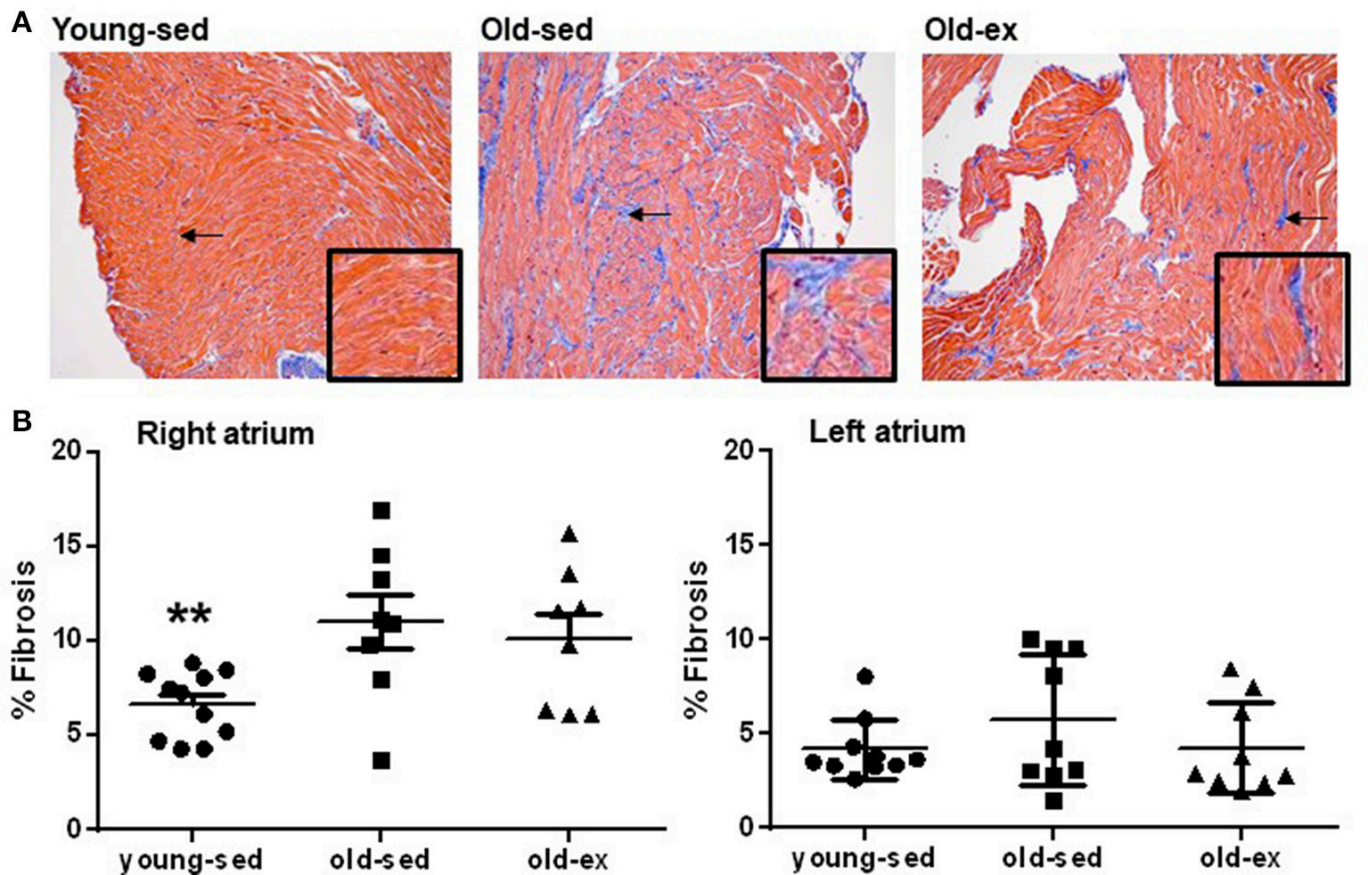
Atrial fibrosis. **(A)** Examples of Massons' Trichrome staining from the right atrium in: Left: Young-sed. Middle: Old-sed. Right: Old-ex animals. The endocardial and perivascular fibrosis seen in the images were excluded from analyses. Insets at higher magnification for better visualization of fibrosis. Arrows denote fibrotic areas. **(B)** Amount of interstitial fibrosis in the right (young-sed *n* = 11, old-sed *n* = 8, old-ex *n* = 11) and left atrium (young-sed *n* = 10, old-sed *n* = 9, old-ex *n* = 9). **p < 0.01 vs. old-sed.

Structural changes were examined from histological slices in each group (example images shown in Figure 4A). In the RA, there was a significant increase in amount of fibrosis in old-sed (*p* = 0.005), which was similar in exercised animals (Figure 4B). There were no significant differences in amount of fibrosis between groups in the LA.

### Immunohistochemistry and western blot

**Figure 5:**
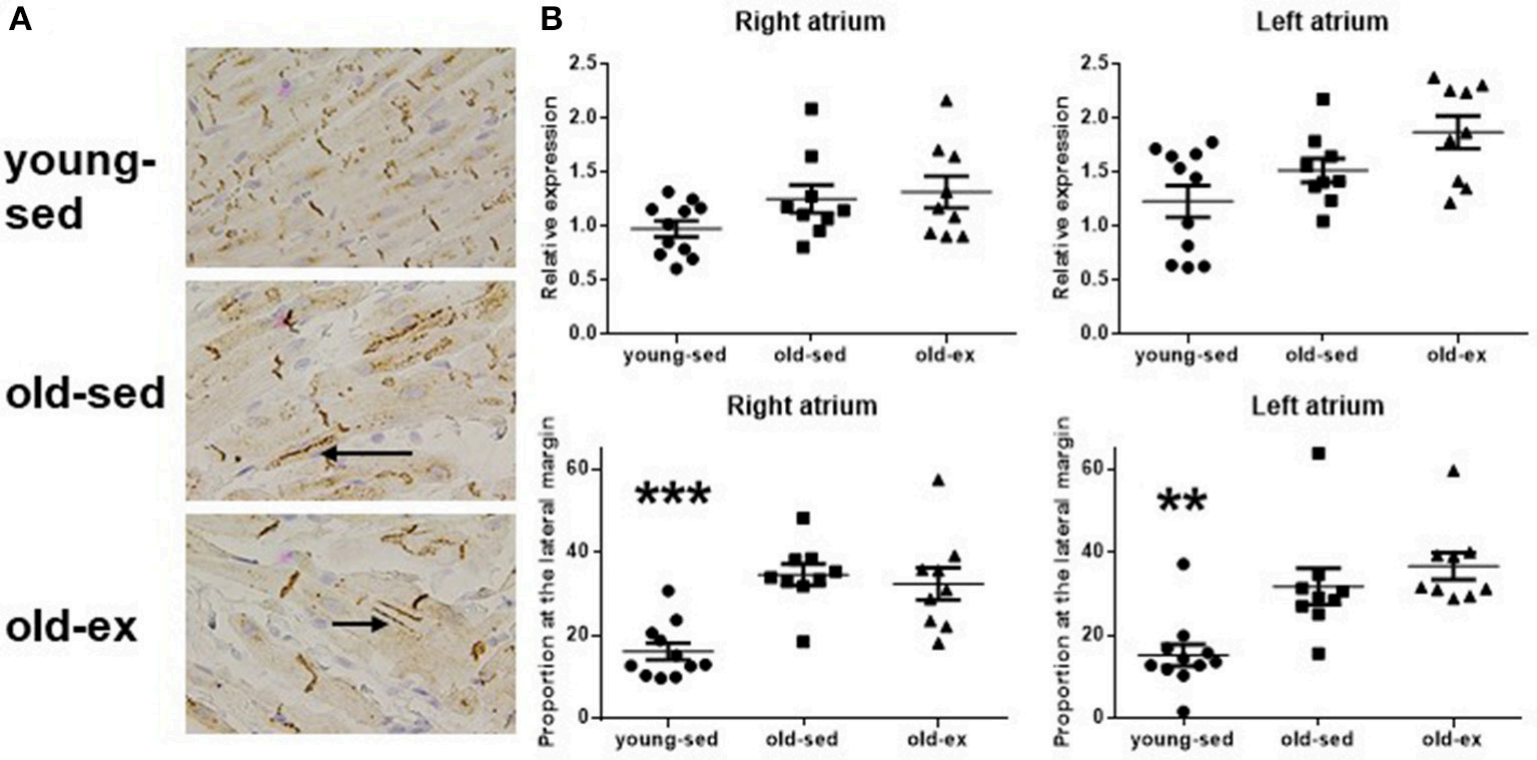
Connexin 43 at immunohistochemistry. **(A)** Photomicrographs of cx-43 stained samples at immunohistochemistry. Upper: Young-sed. Middle: Old-sed. Lower: Old-ex. Arrows denotes lateralization of cx-43. **(B)** Upper: Total cx-43 at the cell surface. Lower: Proportion of cx-43 at the lateral cell margin. Cx-43 = Connexin 43; old-ex (*n* = 9); old-sed (*n* = 9); young-sed (*n* = 11). **p < 0.01. ***p < 0.001 vs. old-sed.

Molecular mechanisms underlying the electrophysiological adaptations to exercise were investigated by examining candidate proteins known to influence CV; namely connexins and the Na^+^ channel. Histological sections from all groups are shown in Figure 5A. There were no differences between groups in total cx-43 area on immunohistochemistry (Figure 5B—upper panels) or cx-43 abundance on western blot (data not shown), but immunohistochemistry showed a significant lateralization with age, for both atria (Figure 5B—lower panels). We performed western blot using an antibody specific to phosphorylation site Ser368 of cx-43, which showed similar results as the general cx-43 antibody. The Ser 279/282 phosphorylated cx-43 antibody did not provide reliable results, as few animals had distinguishable bands in the actual region of the gel. We also attempted to study cx-40 and the Nav1.5 Na^+^ channel, but the tested antibodies did not provide satisfactory staining (data not shown). Only minor expression of cx-40 in rat atria is previously described (Iwasaki et al., 2014).

## Discussion

The present study demonstrates that the increased AF susceptibility with aging was associated with atrial fibrosis, reduced atrial CV and lateralization of cx-43. AIT protected against this age-dependent AF, and was associated with increased atrial CV, prolongation of the AP and reduced AP alternans at higher heart rates, with no detected effects on the increased atrial fibrosis and cx-43 lateralization.

### Changes with aging

Higher susceptibility to AF induction in middle-aged animals was consistent in the study. This is in agreement with findings in humans, where AF prevalence increases considerably from 50 to 60 years of age. Increased susceptibility to AF with age has also been confirmed in several animal models (Sankaranarayanan et al., 2013). It is difficult to directly compare age of rats to human age, but 9–13 months old rats are comparable to middle-aged humans, which were considered appropriate as a starting-point to look at effects of exercise and age, and comparable to the age in which AF risk start to increase in humans (Dun and Boyden, 2009; Sankaranarayanan et al., 2013).

Increased atrial fibrosis was the most prominent atrial structural change in middle-aged animals in the present study. This is in line with clinical studies demonstrating increased fibrosis with aging (Sankaranarayanan et al., 2013). Atrial fibrosis is a strong marker of AF risk, and a direct effect of fibrosis on AF risk has been demonstrated in a mouse model of TGF-β over-activity (Verheule et al., 2004).

Connexins are key components of gap junctions, and studies have shown that dysregulation of connexins, especially cx-43 and cx-40, can be important in AF pathogenesis. In the present study, we saw an unchanged total atrial cx-43 amount, but a significant lateralization in atria of middle-aged rats. Few studies have examined the effect of aging on connexin properties, but changes in phosphorylation state and localization with age have been reported, resulting in less efficient signal propagation between cells (Koura et al., 2002; Sankaranarayanan et al., 2013). One of the few studies that have assessed changes in connexin distribution with age, demonstrated a change from lateral margins to the cell termini in dogs, which was interpreted as AF promoting (Koura et al., 2002). This is contrary to later studies on connexins in AF, where lateralization of cx-43 has been associated with an increased susceptibility to AF, and restoring normal expression and cellular location of connexins at cell termini is shown to prevent AF (Igarashi et al., 2012).

Changes in atrial structure and cell-to cell communication through gap-junctions can both explain the reduced CV seen in old-sed. In addition, excitatory Na^+^-current (I_Na_) is a major determinant of CV (Heijman et al., 2014). We did not measure I_Na_ directly, but the AP rise time is a consequence of I_Na_ activity, and was unchanged with aging. There are limited data on the effect of aging on I_Na_, where some studies find no change with age and some a reduction of I_Na_ at higher stimulation frequencies. The changes with age do however seem to be minor (Sankaranarayanan et al., 2013). From this, changes in I_Na_ seem to be of less importance in AF promoted by aging.

The LA was larger in older rats, but this was proportional to the increase in body weight. However, as LA size has an influence on AF susceptibility, this increase might affect AF risk, despite being related to weight gain. A study in humans has proposed increased LA size to be important for the increased AF risk with obesity and age (Mont et al., 2008; Conen et al., 2013). LV mass was significantly higher in the old-sed group, which was also related to weight gain, and there was a trend toward a reduced EF. These findings are generally consistent with the results of other studies (Boluyt et al., 2004; Lau et al., 2013). When normalizing to body weight, a significant decrease in ventricular dimensions was seen. Unchanged or increased ventricular volumes are generally reported with aging, but those results were not normalized to body weight (Boluyt et al., 2004).

Increased atrial fibrosis and lateralization of cx-43 were the main changes with age in the present study, and are likely important underlying mechanisms for age-associated AF through general and local slowing of CV. Interventions that modify these changes, could be important in AF prevention and treatment. As long-term exposure to AF risk factors results in increased fibrosis, adequate treatment of persons at risk from younger age might reduce the increased AF incidence with aging. Of note, fibrosis levels were only significantly elevated in the right atria. As the left atria were considerably smaller than the right, and only half of the atrial tissue was available for histological analyses, the findings in the current study could be due to methodological limitations. There are also conflicting reports regarding atrial fibrosis with aging in rats in other studies, with some reporting an increase (Hayashi et al., 2002), some report no change (Lau et al., 2013), and one study reporting an increase in the left, but not in the right atrium (Xu et al., 2010). These differences may be due to methodological problems, differences between strains of rats, or differences in age of rats in the studies.

### Changes with exercise

Exercise reduced the age-associated increase in AF susceptibility in the present study. Old-ex animals had a shorter duration of AF and no episodes of sustained AF. There was an increase in CV and a prolongation of APD with exercise, which might explain the reduced AF susceptibility in this group. If maintained at AF frequencies these changes would increase the electrical wavelength of the atria (refractory period times CV) and therefore for the same size of atria make AF less likely (Kettlewell et al., 2013). The reduced AP alternans indicates a reduced heterogeneity of repolarization that may be a by-product of the changes in APD and CV.

Changes in the conduction of electrical impulses and the refractoriness of atrial tissue modulate AF susceptibility. An increase in APD and/or CV protects against AF induction, while local atrial differences in CV and AP properties are AF promoting. There is limited previous data on the effect of exercise on atrial CV. One study showed a decrease with very large amounts of endurance exercise, associated with increased fibrosis, and increased AF susceptibility, which is contrary to our findings (Aschar-Sobbi et al., 2015). Atrial CV and AF susceptibility are shown to be related to connexin properties in several studies (Igarashi et al., 2012). We did not detect any differences in concentration, distribution, or phosphorylation states of cx-43 induced by exercise in the present study. It might be that exercise has no effect on cx-43 properties in the current model, but there might also be differences that we did not unveil. Connexins change their properties within minutes in response to external stimuli, and pacing and periods of ischemia might have induced changes in connexin properties, masking the effects of exercise (Beardslee et al., 2000). We did however try to prevent this by including a 5-min standard pacing protocol at the end of *ex vivo* experiments, to limit individual differences caused by pacing, and the time from cutting the heart down to snap freezing after chamber dissection was ~2 min. There are few other studies examining the effect of exercise on cardiac connexins. An increase in ventricular cx-43 levels with exercise was found in one study (Tiscornia et al., 2014), while another showed unchanged atrial cx-43 levels (Aschar-Sobbi et al., 2015). AP rise time was unchanged with exercise in the present study, suggesting that the differences in CV are not caused by changes in I_Na_.

The present study shows a significant increase in APD with interval exercise, which in part can explain the protective effects of exercise. APD shortening has been shown in multiple studies as a factor predisposing to AF, and is one of the main consequences of long-standing AF (Andrade et al., 2014; Heijman et al., 2014). An increase in APD will generally increase the resistance to arrhythmias caused by reentry mechanisms, but a major prolongation has also been associated with increased APD dispersion, increasing the susceptibility to AF (Heijman et al., 2014). The effect of exercise on APD and atrial effective refractory period (AERP) is varying in previous studies, with an increase, no change, and a decrease all being reported (Guasch et al., 2013; Aschar-Sobbi et al., 2015). Differences in influence on AERP with different types of exercise could explain some of the contradictions in AF susceptibility between studies. A reduced AERP was seen in the study by Guasch et al. and AF susceptibility was increased. In the study of Aschar-Sobbi et al. APD in isolated atrial cardiomyocytes was prolonged, while the AERP *in vivo* was unchanged. Atropine, however, caused a significant prolongation of AERP, which caused an immediate decrease in AF susceptibility.

Exercise increased both atrial and ventricular diameters in the present study, which are well-known adaptations to endurance exercise (Guasch et al., 2013). An increase in atrial size is known to promote AF in the long term in the general population (Mont et al., 2008; Conen et al., 2013), but the AF susceptibility was actually decreased in exercised animals, which can be explained by the electrical remodeling discussed above. Similar findings are seen in humans, where athletes are known to have clearly enlarged atria, but do not exhibit an associated increase in AF risk (Pelliccia et al., 2005).

The reduced AF susceptibility caused by exercise in the present study contrasts the results of Guasch et al. (2013) and Aschar-Sobbi et al. (2015). This can be related to different age of the animals, and different volumes and modes of exercise. In epidemiological studies the response to exercise seems to change with age. The increased AF risk caused by large amounts of exercise is generally seen in younger persons, with no detected increase in those who start to exercise in older age (Drca et al., 2014). This can be caused by younger persons being able to perform exercise at a higher workload, or by different dose-response effects of exercise in older populations. Moderate amounts of exercise can reduce the risk of developing AF (Qureshi et al., 2015), and a favorable effect of AIT in patients with established AF has been demonstrated (Malmo et al., 2016). Several observational studies in humans have shown an increased risk of AF with high volumes of endurance exercise for years (Mont et al., 2009). Different mechanisms are possibly underlying AF caused by exercise, where autonomic changes, with increase vagal activity seems to be important, while increased sympathetic activity seems to be more important in AF in the general population. We did not examine the effects of exercise on the autonomic nervous system, which might be a contributing factor to the results seen. There are also major differences in autonomic activity in *in vivo* and *ex vivo* experiments, which can affect measurements. Both increased parasympathetic activity during deep sleep and increased sympathetic activity during periods of hypotension can be seen under general anesthesia. In *ex vivo* experiments, the influence of the autonomic nervous system is removed.

The animals in the study by Aschar-Sobbi et al. ran for 120 min per day or swam for 90 min, which is a considerable larger workload than the exercise performed in our study. Those animals also had a greater degree of fibrosis than the animals in the study of Guasch et al. which performed exercise for 60 min, indicating that exercise volume is important regarding fibrosis formation and AF risk. Animals in our study performed exercise for 60 min, which is similar to the study by Guasch et al. but the results on AF susceptibility and fibrosis are contradictory. Despite that the animals in our study performed intervals with exercise at high intensity; they had 2-min periods at low intensity every 4th min, which could prevent some of the unfavorable effects on the atria due to a continuous high workload on the heart for 1 h or more. The different results regarding AF susceptibility could imply that a long duration of continuous exercise at moderate intensity is more harmful than repeated short intervals of exercise at higher intensity. This supports findings in humans that large amounts of exercise, both in term of duration and intensity, are required to increase AF risk. Further studies examining different exercise volumes and intensities are needed to provide more insight on this issue.

There is emerging evidence that moderate amounts of exercise and interventions aimed at cardiovascular risk factors are effective in preventing and treating AF. The mechanisms are not clear. Exercise did not prevent, or reverse remodeling caused by aging with no difference in amount of fibrosis or distribution of cx-43, and exercised animals also had an increase in LA size. This indicates other protective adaptations with exercise, and that the increased AF risk due to structural changes in the atria with aging can be offset by electrical remodeling with changes seen in CV, APD and susceptibility to AP alternans. This is in line with the results of Guasch et al. where the AF vulnerability decreased with detraining, despite fibrosis levels being the same (Guasch et al., 2013). A study in humans with AF showed a rapid effect of exercise on AF burden, supporting the finding that other changes than structural are important mechanisms underlying the positive effects of exercise (Malmo et al., 2016). Future studies should further examine the volumes of exercise, both in term of duration and intensity that can induce positive remodeling without causing increased fibrosis and unfavorable vagal changes, and attempt to verify those findings in humans.

### Study limitations

The use of a rat model is a major limitation in generalizing the findings of the study to human AF. Rats have small atria with a prominent auricle and do not have spontaneous episodes of AF. Because the AF episodes were induced, they are not directly comparable to the spontaneous episodes seen in human AF. There are however major methodological, economical, and ethical limitations in using larger animals. The *in vitro* experiments and exposure to ischemia before freezing might have affected results of molecular analyzes, especially the cx-43 properties. Changes associated with aging and exercise are suggestive, but not conclusive for mechanisms of age and exercise-induced effects on AF susceptibility. Interventional studies and studies in larger animal models and humans are needed to verify the importance of our findings. A protective effect of exercise on AF is, however, in line with the results of multiple human studies.

## Conclusions

The increased susceptibility to AF seen in middle-aged rats was associated with atrial fibrosis, dilatation of the LA, lateralization of cx-43 and reduced atrial CV. AIT prevents this increase in AF vulnerability, possibly through increased atrial CV, prolongation of AP and a reduction of AP alternans, with no changes in fibrosis and lateralization of cx-43.

## Author contributions

All authors listed have made a substantial, direct and intellectual contribution to the work, and approved it for publication.

### Conflict of interest statement

The authors declare that the research was conducted in the absence of any commercial or financial relationships that could be construed as a potential conflict of interest. The reviewer MM and handling Editor declared their shared affiliation.
